# Multidisciplinary Antenatal Management of a Late Pregnancy Complicated With Advanced Stage Breast Burkitt Lymphoma - Case Report and a Review of the Literature

**DOI:** 10.7759/cureus.34950

**Published:** 2023-02-13

**Authors:** Zhong Hua, Iqra Ijaz, Muhammad N Shahzad, Duan Yi, Gao Y Hu, Fu X Dong

**Affiliations:** 1 Department of Obstetrics, Luzhou Medical College Affiliated Hospital, Luzhou, CHN; 2 Sichuan Provincial Center for Gynecological and Breast Diseases, Southwest Medical University, Luzhou, CHN; 3 Department of Obstetrics and Gynecology, Holy Family Hospital, Rawalpindi, PAK; 4 Department of Hematology, Luzhou Medical College Affiliated Hospital, Luzhou, CHN; 5 Internal Medicine, Holy Family Hospital, Rawalpindi, PAK; 6 Department of Pathology, Luzhou Medical College Affiliated Hospital, Luzhou, CHN; 7 Department of Radiology, Luzhou Medical College Affiliated Hospital, Luzhou, CHN

**Keywords:** hematologic cancer, multidisciplinary analysis, antenatal chemotherapy, pregnancy, burkitt lymphoma

## Abstract

The multidisciplinary team (MDT) plays a pivotal role in establishing the diagnosis and tailoring treatment for challenging, complicated, rare obstetrical cases. At 28 weeks of gestation, a lady presented with an unresolved unilateral proptosis and sustained severe mastitis. MDT managed the patient at a tertiary care hospital for primary breast Burkitt lymphoma (PBBL). It is a rare and highly malignant condition requiring an aggressive therapeutic approach. Antenatal chemotherapy (ANC) with an aggressive regimen of R-hyper-CVAD/MA was started. A healthy baby was vaginally delivered after completing the second therapy cycle at 32+ weeks, weighing 1.6kg with a good Apgar score. Postnatally, the central nervous system (CNS) prophylaxis was added; after completing eight chemo cycles, our patient remained stabilized for nine months. Unfortunately, due to the refractory and aggressive nature of malignancy, it relapsed, giving an overall survival (OS) of two years. MDT care should be considered at the earliest possible period to expedite the entire process. Positive results can be achieved with timely aggressive treatment and early management of such cases.

## Introduction

Lymphoma in pregnancy is rare, with an incidence of one in 6000 births [1]. Primary breast Burkitt lymphoma (PBBL) is a sporadic, highly aggressive growing tumor in pregnancy with an incidence rate of 0.04%-0.5%. The typical age of onset is 55 years, while onset before the age of 35 is extremely rare. If treated promptly, stages I and II give a favorable prognosis of up to 90%, but in a more advanced stage, when the central nervous system is affected, survival drops to as low as 0-30% [2]. In the literature, there are reports of 21 cases of PBBL linked to pregnancy; 16 of these were diagnosed in pregnancy [3-15], while five were during the breastfeeding period [4,16,17], with an average age of 29.9 years (range 15-42). Eleven of the 16 cases reported during pregnancy occurred in the third trimester, compared to four and one each in the second and first trimesters. The recorded overall survival (OS) was one month in nine, six months in four, and 10 months for one patient, while six patients showed no signs of disease at 12+ months following prompt aggressive treatment. 

Being pregnant while having lymphoma can be challenging, in part because the disease is thought to be incurable and in part because the patient worries that antenatal chemotherapy (ANC) will harm her unborn child. We present the first case of PBBL from China that was found in the third trimester. Our patient knowingly consented to receive ANC with regimen R-hyper-CVAD/MA (rituximab, cyclophosphamide, vincristine, doxorubicin, dexamethasone, methotrexate, and cytarabine). Systemic and/or intrathecal chemotherapy with methotrexate and cytarabine is used for central nervous system (CNS) prophylaxis [18]. Apart from the increased risk of preterm delivery, this regimen is considered safer in the second and third trimesters [19]. For newly diagnosed BL, dose-intensive multi-agent chemotherapy with CNS prophylaxis is preferable, while the R-CHOP regimen is inadequate. Clinical trials are the preferred treatment for relapsing or resistant conditions [20,21].

Thus, the current recommendation for the treatment of Burkitt lymphoma (BL) follows the period of pregnancy in terms of the trimester, the disease nature, whether or not aggressive, and notably, the patient preference [22].

## Case presentation

A young 34-year-old pregnant patient (G4P2A1) was admitted to a tertiary care hospital in China in the 28th week of gestation with unresolved bilateral (B/L) mastitis and extensive right proptosis for three weeks. The unusual primary presentation of an enlarged protruding eyeball was quite a puzzle. The primary emphasis of outpatient therapy was directed toward mastitis and the inflammatory pseudotumor of the eye, which remained unresponsive to treatment. Therefore, In order to fully evaluate her, inpatient hospitalization was recommended. Clinical assessment revealed normal vital signs; an apparent right proptosis without tenderness was noticed, and B/L breasts were diffusely enlarged without redness or palpable lump. The obstetric palpation of the uterus was normal. The lower limbs showed slight physiologic edema. In addition to two cesarean sections (CS) performed 10 and seven years ago with two healthy daughters, the patient disclosed a history of viral hepatitis that was successfully treated ten years prior.

At the time of admission, the patient's white cell count was 7.6x109/l, platelet count was 280x109/l, and hemoglobin was 100g/l. The Epstein-Barr virus (EBV) and human immunodeficiency virus (HIV) viral markers were negative, with only hepatitis B virus (HBV) carrier status. The obstetric ultrasound revealed an intrauterine single active fetus, while on the left side of the uterus, a hypoechoic mass with dimensions 15x10 was observed, mimicking the uterine fibroid. The color doppler ultrasonography (USG) showed B/L inflammatory changes along with swollen lymph nodes (1.3*0.6cm on the right and 1.2*0.5cm on the left) and accessory breast tissue in the axilla.

**Figure 1:**
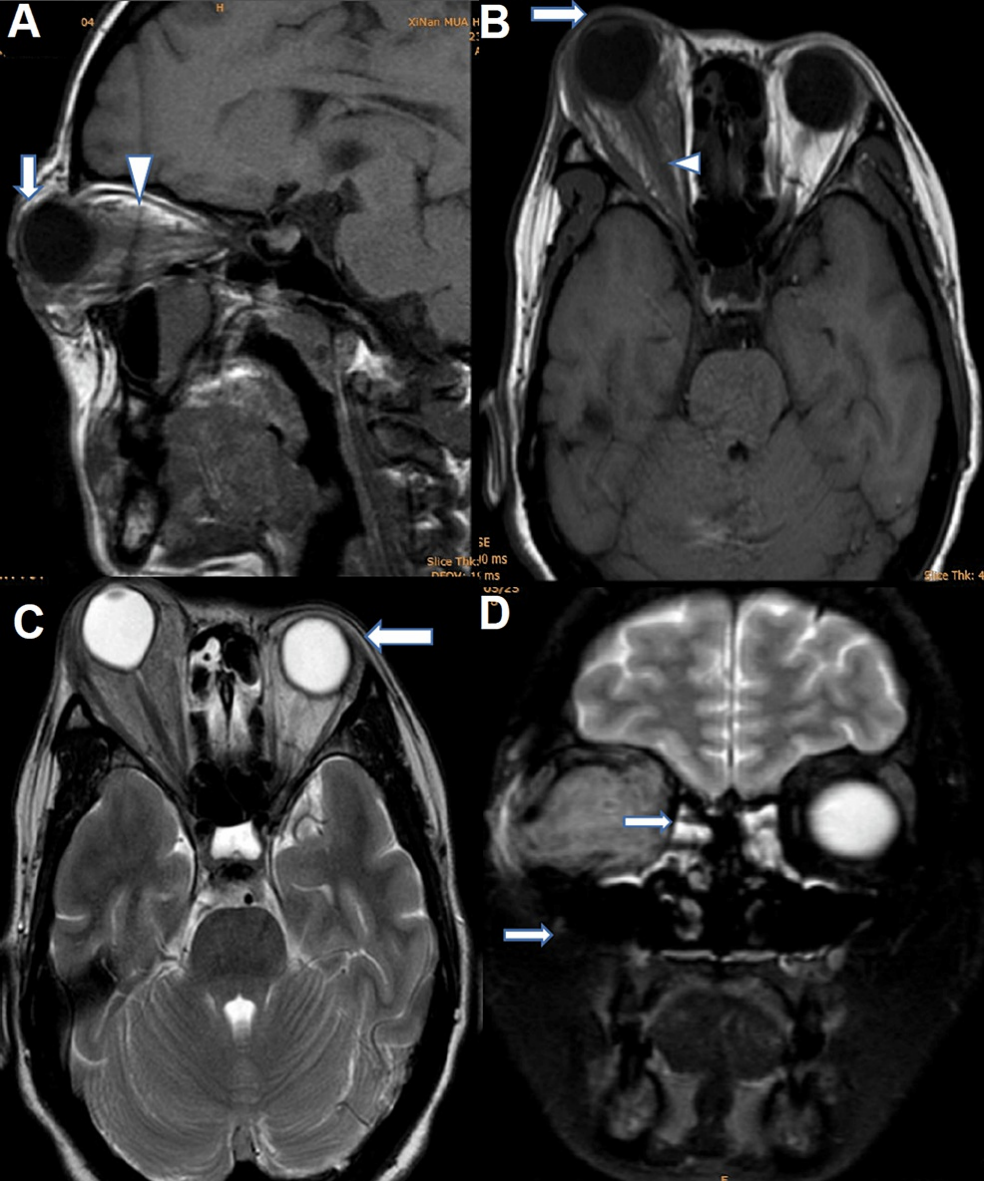
Binocular MRI sagittal, coronal, and axial planes A) Right eyeball prominence with abnormal shape (arrow) due to enlarged anteroposterior diameter, extensively swollen peri and retro-orbital adipose tissue (arrowhead); B) Intact eye ring with normal intra spherical lens, vitreous and anterior chamber morphology (arrow), without thickening in the muscles and optic nerve (arrowhead); C) Left eye shows no abnormality; D) condensed bilateral maxillary, and ethmoid sinuses and effusion are seen on right maxillary and sphenoidal sinuses.

The atypical presentation prompted multidisciplinary team (MDT) discussion (ophthalmology, obstetrics and gynaecology, infectious diseases, breast surgery, neurology) to manage the concealed disease. Relevant examinations proposed by each department were carried out to detect the root cause. The binocular magnetic resonance imaging (MRI) results are shown in Figure 1.

**Figure 2:**
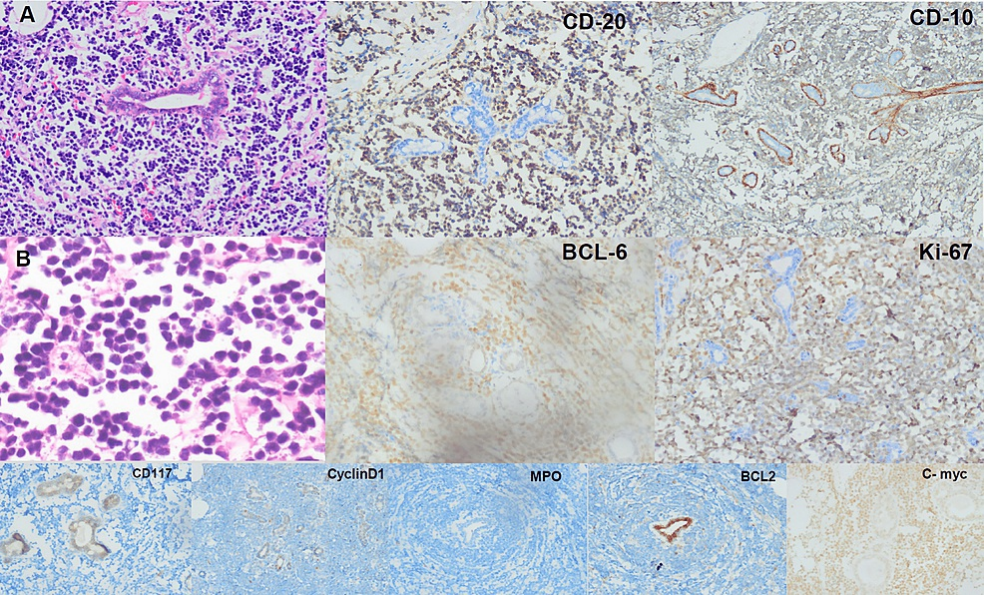
Microscopic, histopathologic, and immunohistochemistry presentation of biopsied tissue A&B) Hematoxylin & Eosin staining ×200, demonstrates that neoplastic lymphoma cells uniformly express CD20, CD10, BCL 6 confirming B lineage (germinal center origin), Ki-67 staining (>70%) of cells defines high proliferative rate and C-Myc staining >90% of cells is another confirmation of the diagnosis of BL. Cells do not express BCL-2; CD117, CyclinD1, and MPO, so other possible neoplasms are ruled out.

Breast MRI requires a prone stance; hence USG was utilized instead, giving the same report. However, It was opted to take a biopsy of the right axillary mass. The microscopic histopathologic and immunohistochemistry (IHC) results of the core biopsy revealed Burkitt cells replacing breast tissue. Highly proliferative and diffuse infiltrates of small round atypical lymphoid cells with relatively uniform and prominent basophilic nuclei with scant cytoplasm and interspersed macrophages demonstrated (starry sky pattern) typical of BL, Figure 2.

**Figure 3:**
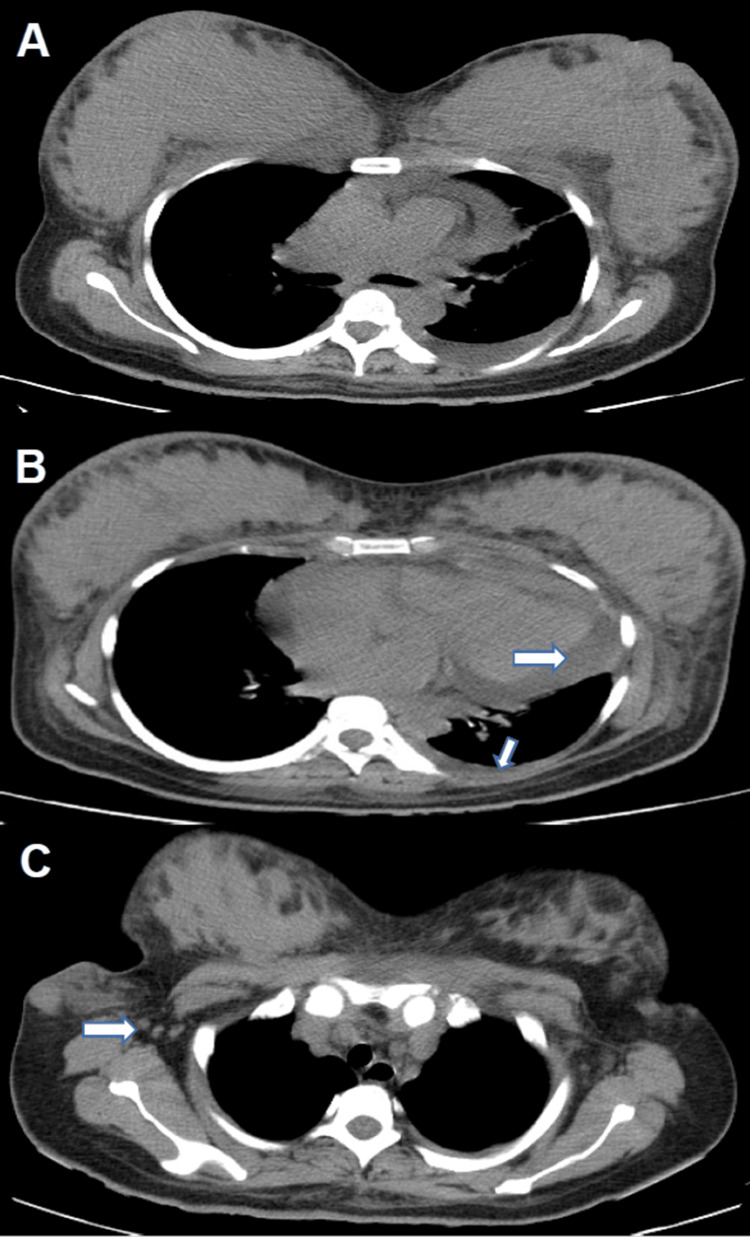
Chest CT A) diffuse bilateral mammary gland inflammation and swelling; B) a small amount of pericardial and pleural effusion (arrows); c. enlarged lymph nodes are seen in the right axillary area (arrows)

To check for metastatic disease and to explore chemotherapeutic options, a chest computerized tomography (CT) scan was ordered (Figure 3).

Given that the patient's situation was rather obvious, the MDT, which included a hematologic oncologist, suggested delaying the baby's birth and commencing the combination chemotherapy right away. Based on the worldwide prognostic index score, the patient was classified as having a high intermediate risk level.

Once the patient was fully informed of her diagnosis and available treatments, she preferred treatment with combined chemotherapy instead of induction of labor or deferring therapy until delivery. Prophylactic entecavir treatment and pretreatment with dexamethasone and polyene phosphatidylcholine were given, and the R-hyper-CVAD/MA regimen was started. The intravenous rituximab 375mg/m2 on day 0 cyclophosphamide 300mg/m2 q12h on days one to three, mesna 600mg/m2 on days one to three, vincristine 2 mg on days four and 11, doxorubicin 50 mg/m2 on day four, dexamethasone 40mg po on days one to four and 11-14, methotrexate 1000mg/m2 for 24 hrs on day one, tetrahydrofolate first rescue dose 50mg then 15mg q6h for a total of six doses, cytarabine 3mg/m2 q12h on days two to three. The methotrexate and the cytarabine were added after the baby's birth.

As soon as the therapy was initiated, the patient responded well, and proptosis and breast swelling reduced remarkably. Due to ongoing chemotherapy, pregnancy-related symptoms were aggravated (shortness of breath, palpitations). After the second chemotherapy cycle, the patient began to experience neutropenia. She proceeded into natural spontaneous uterine contractions (preterm labor), and labor progressed quickly, as the gestational age was only 32 weeks, so the patient spontaneously delivered vaginally a healthy baby weighing 1.6 kg with Apgar scores of eight and 10 at one and five minutes, respectively. Due to prematurity, the newborn was sent to the neonatal intensive care unit (NICU).

**Figure 4:**
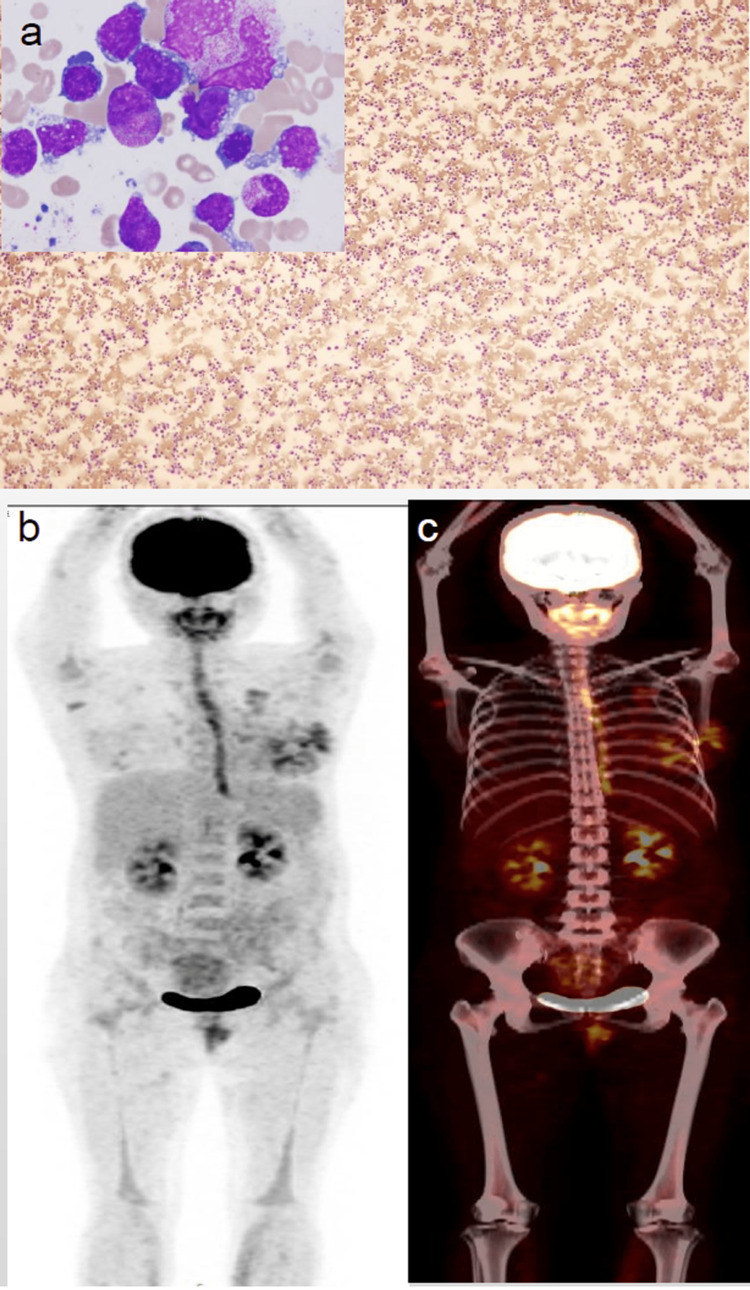
Positron emission tomography-CT and histopathology A) Typical Burkitt lymphoma cells are distributed in the bone marrow biopsy film. Enlarged cell bodies are seen with grayish-blue cytoplasm and a large deviated nucleus. Chromatin is rough and uneven. Several vacuoles of different sizes are seen; B&C) The whole body maximum intensity projection (MIP) image shows nodular uptake of agent in lungs and diffuse uptake in the esophageal wall, medial gastrocnemius, the spine, pelvis, long bones; upper femur right side; cervical, thoracic, and lumbar vertebrae show low degree degeneration

Methotrexate was added on day one for CNS prophylaxis, followed by R-hyper-CVAD/MA regimen. The therapy worked well, and symptoms gradually improved. To further prevent tumor lysis syndrome (TLS), the patient was given fluids, sodium bicarbonate to alkalinize the urine, febuxostat to lower uric acid, furosemide to induce diuresis, and hypertonic sugar with insulin. Reduced glutathione injection and human serum albumin were added for raised liver enzymes. The recombinant human granulocyte colony-stimulating factor (G-CSF), antibiotics, and blood products were used to manage the post-chemotherapy myelosuppressive period of neutropenic fever. The patient responded well to the treatment, completed eight recommended chemotherapy cycles, and remained stable for nine months. However, later, on follow-up, the patient complained of feeling sick and having body aches; therefore, a positron emission tomography (PET)-CT examination revealed a refractory BL relapse with a distant spread, and bone biopsy results showed the same results (Figure 4).

Further treatment was required, but the patient refused and requested only symptomatic relief. She had completed eight cycles of systemic chemotherapy, but unfortunately being very aggressive nature of the tumor with abrupt relapse and metastasis made it challenging and required further chemotherapy; although the patient was urged to continue her therapy, she stopped after six weeks and passed away with a two-year OS owing to multi-organ failure.

## Discussion

Lymphoma type, signs, and symptoms during pregnancy are not different from non-pregnant women. Currently, there is no evidence that pregnancy accelerates the development of lymphoma; however, lymphoma follows its natural course of development and affects the progression of pregnancy, warranting its timely treatment. Since there are few data demonstrating that this pathological kind seems more malignant during pregnancy, the prognosis is poor. Misdiagnosis is common, typically brought either by unusual clinical manifestations or by considering physiological changes related to physiological changes that make it difficult to distinguish from lymphoma hyperhidrosis [20,21]. Malignant lymphomas can develop as a result of certain risk factors, such as ionizing radiation and viruses like EBV and HIV [3]. Although our patient had a history of HBV infection and had sufficient treatment, it is highly unlikely that this chronic illness served as a risk factor for the current attack.

Pregnancy-related BL needs to be identified quickly, and it needs to be treated aggressively right away. It progresses rapidly and can lead to death within weeks or months if not treated aggressively; CHOP-based regimens are ineffective and should be treated with intensive chemotherapy considering a therapeutic regimen consisting of high-dose cyclophosphamide. BL has shorter chemotherapy intervals than other diseases because of its high degree of malignancy; Chemotherapy is given once every one to two weeks, and the condition is assessed at two to four cycles and uses six to eight cycle courses of treatment.

Pregnancy should be terminated for patients with limited early disease with added radiotherapy (RT) in the first half. The antenatal combination chemotherapy can be started in the second half, while RT is added after the fetus is born [20]. This is a case of third trimester that was diagnosed timely with the immense help of a multidisciplinary approach. Even though termination of pregnancy by cesarean section was an option, as the prognosis of the premature newborn was good, our patient preferred antenatal chemotherapy, which positively affected the disease.

On the other hand, several imaging examinations are restricted to avoid fetal radiation exposure; the best opportunity for therapy is lost as a result of further diagnostic delays. Therefore, it is indicated that pregnant women with inexplicable fever, tiredness, a tendency to hemorrhage, unusually enlarged blood cells, as well as developing anemia, have further necessary tests, including bone marrow testing. The standard of prenatal screenings needs to be raised for early disease diagnosis in order to provide prompt treatment.

Labor was once thought to need to be induced right away if lymphoma was discovered during pregnancy. The main justification for this was that active therapy with cytotoxic drugs during the first trimester could have negative consequences on the fetus [23]. Preterm delivery, low birth weight, fetal or neonatal death, and significant bone marrow suppression as a result of disease and therapy that endangers the mother and the fetus at any time are complications for the second and third trimesters [8]. There is yet no conclusive evidence that labor induction improves prognosis. Therefore, if the condition itself is not necessary, it is worthwhile to continue the pregnancy through the middle and late phases of pregnancy while being well monitored [15].

Our patient had significant respiratory depression, but with continued therapy, her condition stabilized. The author prefers to end the pregnancy after organ malfunction manifests in the middle or late stages of the pregnancy. Despite potential drawbacks, the curative therapy's overall value is obvious; hence, it is recommended to administer ANC, and pregnancy should be prolonged as long as it can be and taken near to term gestation.

## Conclusions

Managing lymphoma in pregnancy poses significant diagnostic and treatment challenges, so an MDT is crucial in managing such cases. Therefore, it is advised to consider this aspect promptly for decision-making. Additionally, treatment-related decisions require weighing several feto-maternal risks posed by the disease itself and furthering its treatment on an individual, case-by-case basis.
